# Gun-shot Wound, with Removal of Rim of Acetabulum and Dislocation of Femur—Recovery

**Published:** 1866-05

**Authors:** J. F. Miner


BUFFALO

### Medical and Surgical Journal.

VOL. V. MAY, 1866. No. 10.

ART. I.—*Gun-shot wound, with removal of rim of acetabulum and dislocation of femur—Recovery.* By J. F. Miner, M. D.

The following remarkable and interesting case of recovery after gun shot wound involving the hip-joint is so rare, and in result so unusual, that an account of it may be interesting and valuable as an additional case of recovery among the rare ones which have been observed by military surgeons. Perhaps the whole surgical history of the Rebellion will not furnish a parallel.

Lieut. Col. James Strong of the 38th N. Y. V., now Brigadier General of the Veteran Reserve Corps, was wounded May 5th, 1862, at the battle of Williamsburg, Va. The ball entered a little below the anterior superior spinous process of the illium, and made its exit near the outer margin of the sacrum. The ball passed deeply, and fractured in its course the rim of the acetabulum, which was removed an inch and a half in length and of a diameter sufficient to show that the whole upper rim had been carried away.—This fragment of bone was removed from the wound at the dressings made while in the hospital, where he was carried after having lain on the field for some hours. Surgeon A. J. Berry first dressed the wound and removed fragments of bone and portions of pants and drawers. The wound was very large, and a thorough examination could be made by the easy passage of the finger. After a stay in military hospital of eight days, he was placed on board ship and taken on his way home by his brother and assistants, coming the whole distance upon a stretcher made for the purpose, and suffering the greatest pain from every motion of the boat or car. By force of great resolution and courage his home in Buffalo, situated upon the bank of the Niagara, was reached after a painful journey of five days. The belief that recovery would be impossible in a military hospital or even in a warm climate, under most favorable circumstances, prompted an undertaking which could never have been made from less urgent inducements. On his arrival the wound presented a healthy appearance, and the constitutional disturbance was slight, considering the nature of the injury. The leg was partly flexed upon the thigh, and the thigh upon the body, the whole drawn internally to considerable extent. In this position it was fixed; it could not be moved in the slightest degree without producing pain, which was absolutely unindurable. The constitutional disturbance at length became very great, and the question of life rather than position of the leg engrossed all attention. Fragments of clothing and speculæ of bone were at various times extracted or washed from the wound, while great quantities of pus constantly issued. Chills, profuse perspiration, rapid pulse, great prostration, with total loss of appetite, make up the main features of the case, the severe and continual pain overshadowing and covering all other symptoms—pain which it was impossible to allay. In this condition of extreme distress and uncertainty of life he continued without great change for about eight or ten weeks, when he gradually and very slowly commenced to improve, both in his general condition and in the appearance of the wound, until after a few weeks more he could be moved from one side of the bed to the other, and his comfort promoted by change of position.

When at length it became apparent that his recovery was probable, the question of the condition of the leg was again agitated, and efforts were early made to extend it, and obtain motion in the knee-joint. Extension and counter-extension under the influence of chloroform was adopted, and persevering efforts made to obtain as favorable results as possible. Nothing, however, can be said to have been gained by these efforts other than to have obtained greater motion in the knee-joint, the hip being made neither better or worse. Abscesses occasionally formed in the site of the old wound, and continued to do so for eighteen months or two years, each time inducing great constitutional disturbance, sometimes so great as to excite fears of his life.

The General has been on duty for the last two years, and for most of the time has enjoyed a comfortable degree of health. The present condition of the thigh will complete the main features of a case, which, to avoid being tedious, has been recited more in aggregate than in detail.

The thigh is shortened three and a half inches; the head of the femur rests upon the illium above the acetabulum, and a complete and bony anchylosis exists; the knee is drawn inwards and the joint has good motion. The weight of the body can be borne upon that leg with comfort, and the twisting of the pelvis and the extension of the toes compensates for the shortening and stiffening in a wonderful degree, so that he walks with, or without a cane, with but slight disability, considering the severity of the injury and the unnatural condition of the articulation.

The gravity of gun-shot wounds involving the larger joints has been observed by physicians, both civil and military; the danger from such wounds varying with the extent of injury, the destruction of the tissues, size and kind of joint, and state of health and circumstances of case after injury. It was formerly supposed that injury admitting air to the synovial structures was very dangerous, but this view is not now so universally adopted, and probably the air thus admitted is not of itself so important. Such injury of the small joints are not very dangerous, but of 65 cases of gun-shot wounds of different joints related by Alcock, 33 only recovered, 21 of these with loss of limb, leaving only 12 recoveries without amputation out of the 65 cases. Gun-shot wounds involving the hip-joint have always been regarded as fatal, and very few cases can be cited as exceptions to this general rule. Gun-shot wounds of all large joints are dangerous to life and limb, especially so when the articular extremities of bones are involved in injury; the knee-joint is scarcely less important in results of injury than the thigh, and the most experienced military surgeons have not seen a case of recovery without amputation, when attended with severe injury of the bones.

A gun-shot wound such as related, carrying away the rim of the acetabulum and allowing the muscular contraction to dislocate the femur, drawing the head upwards and backwards, must be a very severe and dangerous injury. If such cases have occurred they have more generally died from either shock, hemorrhage, or inflammatory action and traumatic fever, within a few days — have rarely continued under observation long enough to accurately determine their nature, or the results of imperfect recovery. That gun-shot wound of the head of the femur would be more serious than of the rim of the acetabulum is not improbable, but recovery from either is sufficiently infrequent to make every case which does occur, of the greatest importance in determining probable results of similar injuries.

